# Senescent tumor cells building three-dimensional tumor clusters

**DOI:** 10.1038/s41598-018-28963-0

**Published:** 2018-07-12

**Authors:** Hyun-Gyu Lee, June Hoan Kim, Woong Sun, Sung-Gil Chi, Wonshik Choi, Kyoung J. Lee

**Affiliations:** 1 Department of Physics, Korea University, Seoul, 02841 Korea; 2 Department of Anatomy, Korea University College of Medicine, Seoul, 02841 Korea; 3 Department of Life Sciences, Korea University, Seoul, 02841 Korea; 4 Center for Molecular Spectroscopy and Dynamics, Institute for Basic Science, Seoul, 02841 Korea

## Abstract

Cellular senescence, a permanent cell-cycle arrest, is a common yet intriguing phenomenon, in which its beneficial significance for biological organisms has only begun to be explored. Among others, senescent cells are able to transform tissue structures around them. Tumor cells, whose hallmark is their ability to proliferate indefinitely, are not free from the phenomenon. Here, we report a remarkable observation where senescent cells in a dense mono-layer of breast cancer colony act as aggregating centers for non-senescent cells in their vicinity. Consequently, the senescent cells actively form localized 3D cell-clusters in a confluent 2D tumor layer. The biophysical mechanism underpinning the surprising phenomenon primarily involves *mitotic cell-rounding*, dynamic and differential cell attachments, and cellular chemotaxis. By incorporating these few biophysical factors, we were able to recapitulate the experimental observation via a cellular Potts Model.

## Introduction

Cellular senescence is a common phenomenon in biological organisms in which a proliferating cell enters into a complete growth arrest and dramatically expands its volume (typically, in a form of *fried egg* in two dimensional substrates). The origin of this cell state has been intensely investigated; yet its underlying mechanism is far from being clear^1,2^. Importantly, a senescent cell interacts with its neighbors via a large number of secretions collectively termed as senescence-associated secretory phenotypes, or SASPs. These secretory phenotypes are known to be involved in a variety of biological processes many of which have negative impacts on an organism. For example, pro-inflammatory cytokines and chemokines that stimulate growth of nearby malignant tumor cells are among them^3,4^. The accumulation of senescent cells is also associated with adverse effects in a more organismic level, such as age-related diseases^5^. Particularly, they can also promote tissue remodeling. For example, some senescent cells secrete proteases that degrade extra-cellular-matrix, making nearby tissue structure softer, thus promoting the invasion of cancer cells^6–8^. Beneficial effects, on the other hand, of senescent cells are also discussed lately. SASP includes proteins that contribute to embryonic patterning^9,10^ as well as wound healing^11^. Nevertheless, the exact nature of how these tissue-remodeling effects are biophysically orchestrated by SASP has much to be explored, especially at the scale of an individual cell to a tissue.

In this paper, based on *in vitro* cultures of monoclonal cell line MDA-MB-231 (widely used, highly malignant breast cancer cell line), we carefully analyze the emergence of senescent cells from the initial seeding and their interaction with neighboring non-senescent cells. Surprisingly, even the immortalized tumor cells were found to be susceptible to senescence^12^. More intriguing was the fact that the senescent MDA-MB-231 cell acts as a center of attraction for adjacent tumor cells, initiating a morphological transition from an initially two-dimensional (2D) colony of mono-layer to a three-dimensional (3D) cell cluster. We view that the transition presents a clear *in vitro* example of how senescent cells could be involved in tissue remodeling. We also provide a heuristic explanation on the observation via a computer model integrated with only a few essential mechanisms. The cellular Potts model (CPM), which at its base operates on Metropolis kinetics, is aimed at reproducing such biophysical processes as the conservation of a cell’s volume, mitotic cell-rounding (consequently, the dynamic strength of cell-environment adhesion), and chemotactic movement of a cell.

## Experimental Results

**Figure 1:**
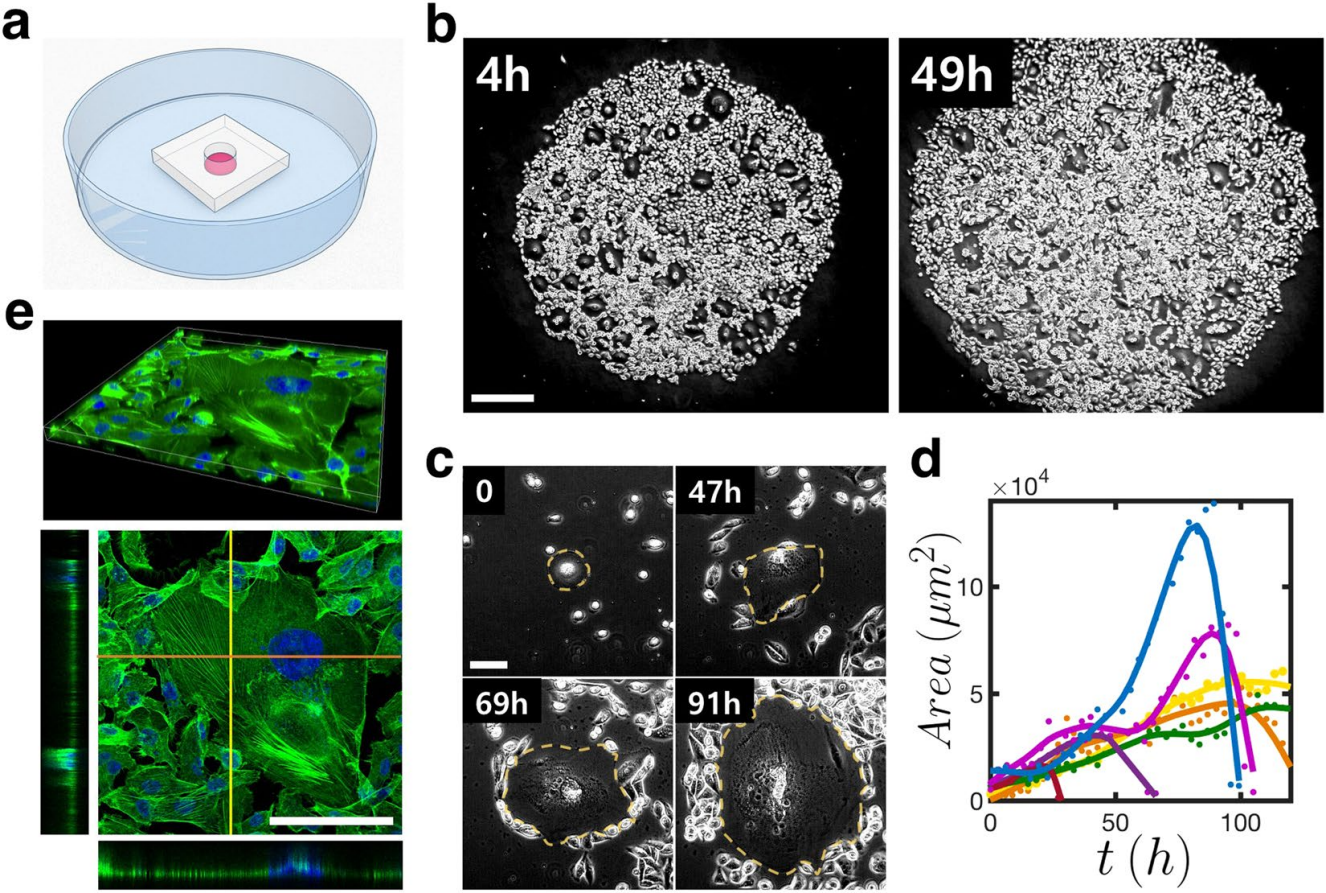
Spontaneous emergence of cellular senescence in populations of MDA-MB-231 breast cancer cells. (**a**) Schematic diagram illustrating a small polydimethylsiloxane (PDMS) well in a culture dish. Detached cells (red) are put into the well; the PDMS is removed after 4 5 hours of planting. (**b**) Phase-contrast snapshot images taken at two different times after the initial seeding; ‘holes’ are the areas occupied by senescent cells. (**c**) Close-up views of an exemplary cellular senescence (outlined by dashed lines) developing in time. (**d**) The growing (followed by apoptosis) areal sizes of several different senescent cells. (**e**) An exemplary confocal 3D immuno-stained image of a senescent cell showing the distribution of filamentous actin marker phalloidin (green) and nuclei marker Hoechst staining (blue). Each side view shows a slice cut in z-direction at the highlighted locations (stretched three times for a better visualization). The 3D image consists of a confocal z-stack of 49 images. [scale bars: (**b**) 500 *μ*m; (**c**) and (**e**) 100 *μ*m]

In a uniformly plated confluent mono-layer of MDA-MB-231 cell culture (initially, onto a disk area of diameter 2 mm; see Fig. 1a; more details in Methods), a number of senescent cells randomly emerge as the whole population grows in time (Fig. 1b). They can be easily identified by their ‘fried egg’ morphology (Fig. 1c). Body of a cell entering into the senescent state expands laterally over days (Fig. 1c) to occupy a huge area even within a quite confluent population. The area occupied by a fully developed senescent cell can notably vary from one to another but is generally very large, sometimes as large as 1.4 × 10^5^ *μ*m^2^ (see Fig. 1d)–which is approximately three orders of magnitude larger than that of a typical non-senescent cell. On the other hand, the senescent cell’s body is as thin as ~2 *μ*m (see the two side views in Fig. 1e). The body is structurally well-maintained by a dense network of f-actin (see the top view in Fig. 1e). Ceaseless spatiotemporal wave is present all over the body and directed towards the core, until the cell abruptly bursts to end its metabolic processes.

**Figure 2:**
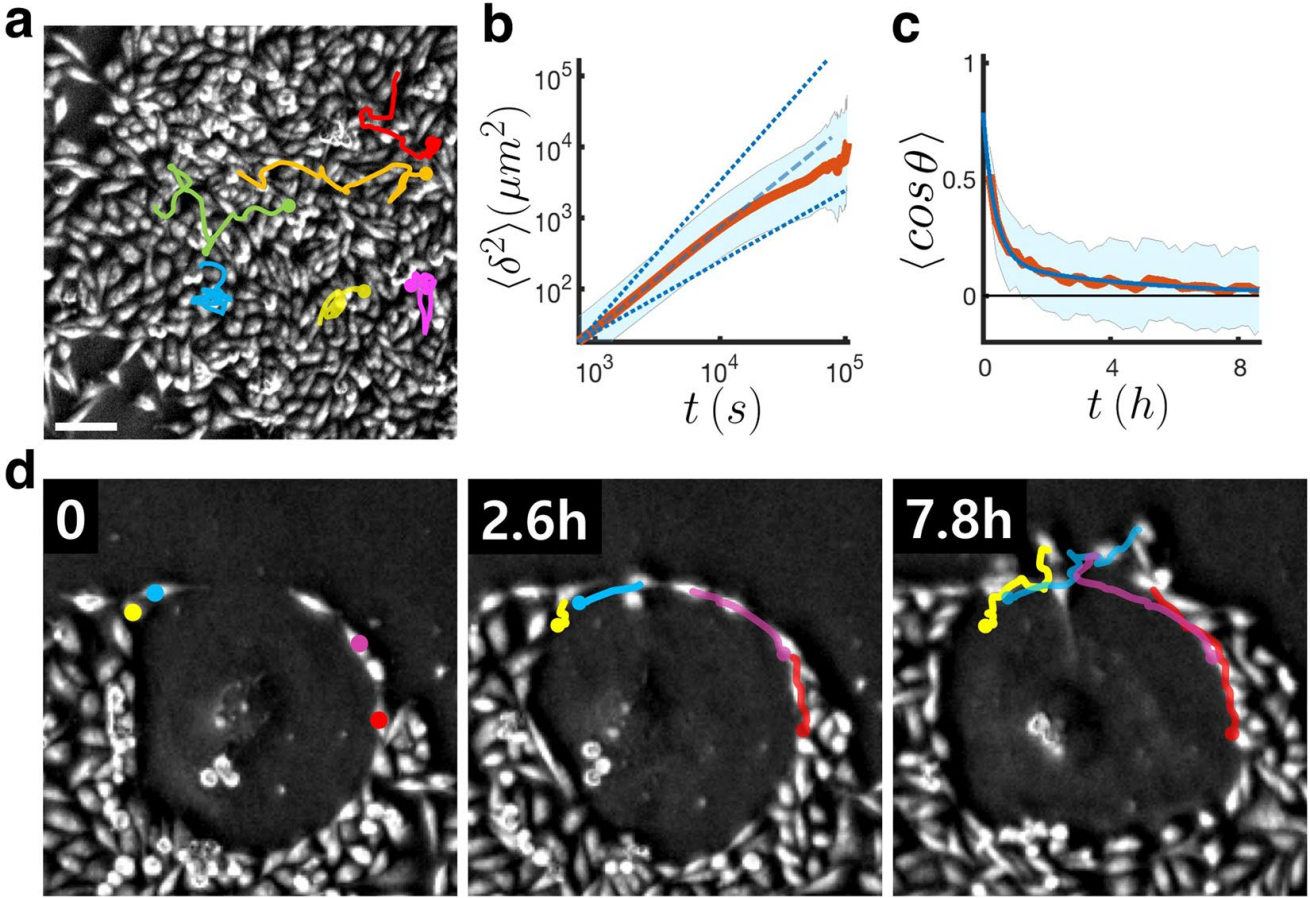
Motile behaviors of MDA-MB-231 cells within the cell colony. (**a**) Cells percolating within a confluent population and (**d**) Cells exhibiting haptotactic movements along the boundary of a senescent cell body (see Supplementary Video S1). (**b**) is the log-log plot of the average of mean-squared displacements vs. time interval (n = 42) illustrating the super-diffusiveness of the cells in (**a**). (**c**) 〈cos *θ*〉 vs. *t* (red line) for the same *n* = 42 cells and its fit to the function $A{e}^{-t/\tau }+B{e}^{-t/{\tau }_{0}}$ (blue line) with *τ*_0_ = 0.38 hr, *τ* = 4.56 hour, *A* = 0.14, and *B* = 0.64. The shades in (**b**,**c**) represent the standard deviation at each *t*. The scale bar of 100 *μ*m applies to all phase-contrast images in this figure.

**Figure 3:**
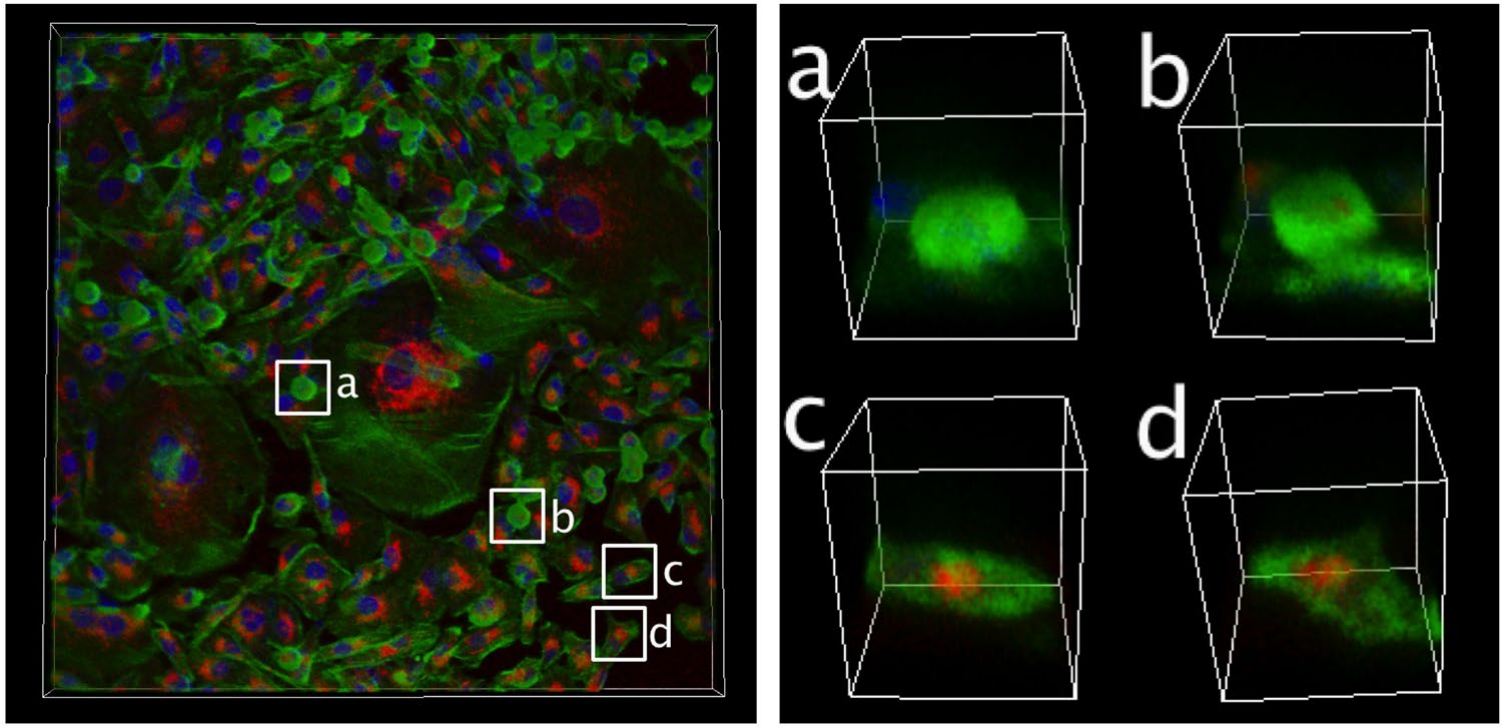
Confocal immuno-stained image of MDA-MB-231 cells, highlighting cell-rounding. The insets (**a**,**b**) are two examples of a cell undergoing mitotic cell-rounding during which the level of E-cadherin expression is diminished significantly, as compared to that of normal cells (for examples, **c** and **d**). The level of E-cadherin expression for the huge senescent cells, which barely moves, is also high near the nuclei. Distribution of filamentous actin marker, phalloidin (green), E-cadherin marker (red) and nuclei marker, Hoechst (blue), is shown.

Neighboring non-senescent MDA-MB-231 cells stay assembled with high fluidity due to the enhanced motility derived from cell-cell contact interaction. Accordingly, even within a confluent population an individual cell can travel distances (Fig. 2a) much greater than the size of a typical cell: In fact, the cells are super-diffusive with the anomalous exponent *α* = 1.42 ± 0.06 for the mean-squared displacement 〈*δ*^2^〉 ~ *t*^*α*^ (Fig. 2b). Their trails within the population may be considered as a worm-like chain having a directional persistence. The mean directional persistence time *τ* = 4.6 hr (*n* = 42) can be estimated by fitting 〈cos *θ*(*t*)〉 to a two-tiered exponential function $A{e}^{-t/\tau }+B{e}^{-t/{\tau }_{0}}$, where *θ*(*t*) is the angle between two directions to which a given cell is heading at two different times separated by *t* (Fig. 2c). The behavior of normal MDA-MB-231 cancer cells within a densely packed domain suggests the jamming transition discussed in ref.^13^. On the other hand, a fully expanded senescent cell barely moves in the confluent situation and exhibits a quite unusual interaction with nearby non-senescent tumor cells as shown in Fig. 2d. Cells in direct contact with the senescence show higher directional persistence along the boundary (Fig. 2d), allowing them to circle around the senescence. Yet, these cells neither step onto the thin surface of the senescent cell, nor stray away from it to the empty space (see Supplementary Video S1), resembling a microglia moving haptotactically along the network of trails^14^. In addition, through studying immuno-stained image of the cell type, we concluded that observed affinity between the senescent and non-senescent cells cannot be attributed to the prominent cell-cell adhesion protein, E-cadherin, because of the lack of E-cadherin at the boundaries of the cells (colored red in the top-view 2D image in Fig. 3).

The behavior of non-senescent cell at the senescent cell boundary seems closely associated with membrane ruffle dynamics. The pre-existing ruffle activity of both senescent and non-senescent cells disappear at the contact line where non-senescent cell (temporarily) adhere and contract upon collision as in the well-known phenomenon coined as “contact inhibition of movement”^15^ (see Supplementary Fig. S1). Then, the non-senescent cell prefers to move along the boundary of senescent cell with its new ruffles forming mostly in the direction of movement (see Supplementary Fig. S2): The ruffle activity seems quite suppressed along the contact line. We believe that the tangential movements of non-senescent cells are a consequence of (1) active crawling of non-senescent cells, (2) the fact that these cells prefer to adhere each other than stand alone in the medium and this preference should be mediated by haptotactic interaction, which can involve a nexus of actin cytoskeletal and adhesion molecule dynamics as reported earlier^16,17^, and (3) for the non-senescent cells the dorsal surface of the senescent cell is less adhesive than the tissue culture substratum.

**Figure 4:**
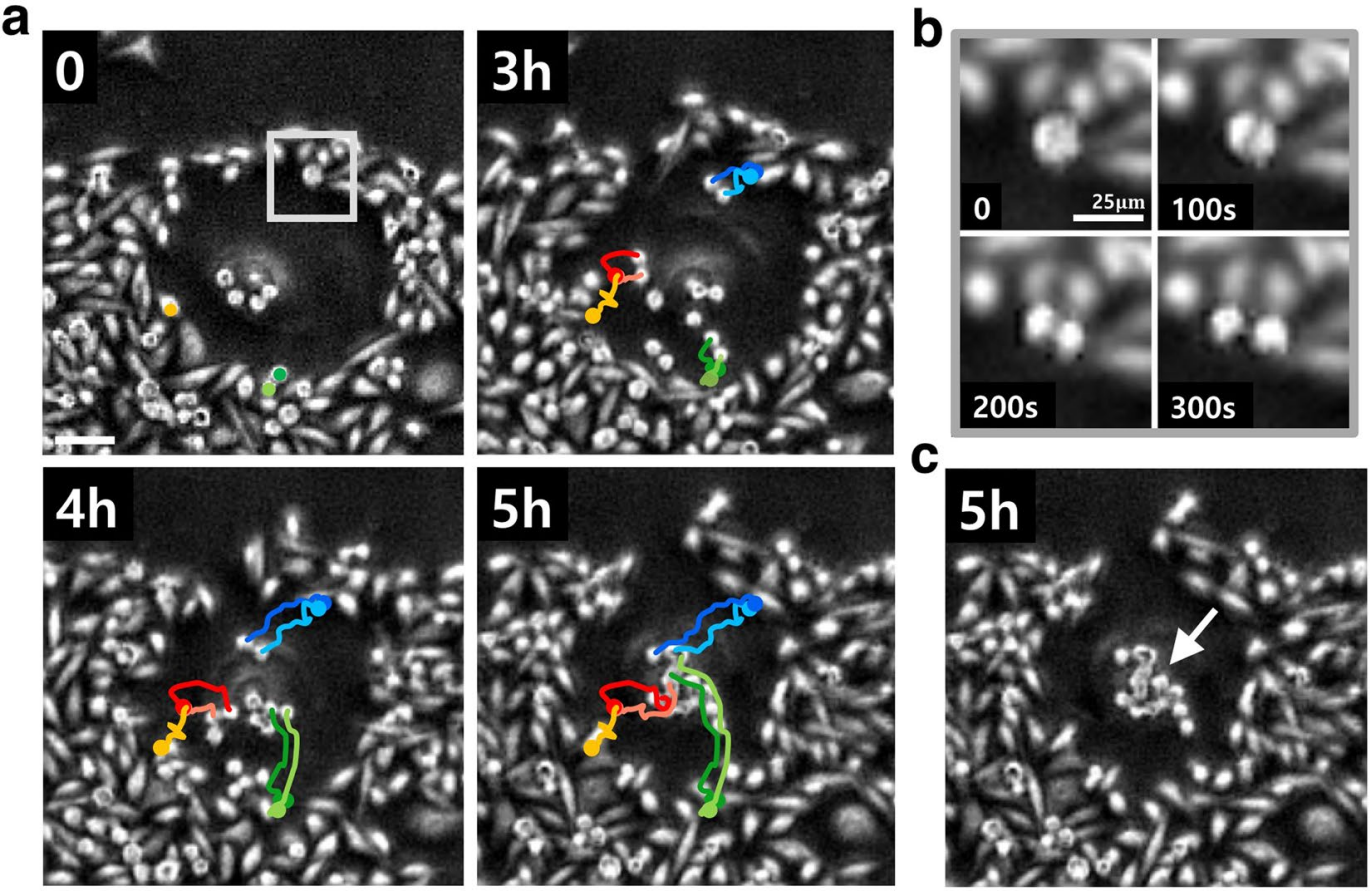
Migration pattern of MDA-MB-231 daughter cells on the body of a senescent cell. (**a**) The sequence of snapshot images shows cells undergoing cell-rounding, all of which are newly replicated daughter cells, migrating towards the senescent core (mean radial velocity: 38.7 *μ*m/hr) (see Supplementary Video S2). (**b**) A sequence of close-up images showing a process of cell division and, the simultaneous mitotic cell-rounding [square-boxed area in (**a**)‘s first sequence]. (**c**) A snapshot image of a senescent cell culminated with a small cell cluster (marked by an arrow) attached onto its core. (scale bar: 100 *μ*m).

Any proliferating cell population on a flat substrate is jammed if its areal expansion cannot create enough space for the dividing cells. The 2D jamming leads to either a halt in further proliferation^18^, or a multi-layered cell population^19^. The MDA-MB-231 breast cancer cell colony forms clusters of cells out of the 2D plane as a consequence of continued replications (approximately at every 28.7 hr) upon the jamming. Intriguingly, a senescent cell in the population can serve as a source of premature multi-layer transition: The senescent cell gathers newly replicated cells, as well as the cells that are about to divide, to its center by transporting them on its body. This action begins when an adjacent cancer cell loses its adhesion to the floor while entering into a process known as mitotic cell-rounding^20^. Cells undergoing rounding are marked as **a** and **b** in Fig. 3. These cells are distinguished by the concentrated actin filaments (green) all over the surface of its spherical body (relative to the cells not undergoing cell-rounding, labeled by **c** and **d** in Fig. 3). Also visible is the notable height of the rounded cell in Fig. 1e (left side view). In a confluent population, the process of mitotic cell rounding brings much portion of dividing cell out of the 2D plane before the actual mitosis takes place. The orientation of the axis of cell division is generally co-planar to the 2D substrate plane, but it can be also affected by its neighboring environment. Interestingly, the daughter cells with the shape of a dumbbell (see Fig. 4a,b) climb over the senescent cell’s boundary that exists in a form of continuous ruffle, and migrate towards the core (see Fig. 4a and Supplementary Video S2). A cell cluster is formed as a result of series of these events all over the perimeter of the senescent cell (marked by an arrow in Fig. 4c). The migratory behavior of non-senescent cells over the dorsal surface of senescent cell body are noisy but directed. Almost every (>90 %, *n* = 20) cell undergoing replication at the boundary of a senescent cell eventually ends up at the core and participates in the formation of cluster.

Is crowding around the senescent boundary essential for dividing cells to migrate to the core? The answer is no. Neighborhood crowding may facilitate the chemotactic drive imposed by the adjacent senescent cell. But, it seems not essential for the directed migration of newly divided cells towards the core, since we observe the same phenomenon frequently in non-crowded environment (see the new Supplementary Fig. S3). Moreover, sometimes only one of the two daughter cells migrates towards the core while the other remains or rejoins the bulk population (see the new Supplementary Fig. S4): There seems to be environmental or stochastic factors involved in association with cell-to-cell adhesions.

The observed phenomenon has an interesting analogy with that of aggregating Dictyostelium discodium (Dd) amoebae^21^, which upon starvation produce a 3D cell mound from an initial 2D cell layer at its pacemaking center that is just one of the cells from the population. It is well-known that cyclic adenosine monophophate (cAMP) serves as a chemo-attractant for chemotaxing Dd. Likewise, there are SASPs, epidermal growth factor as an example, which are identified as chemo-attractants for tumor cells including MDA-MB-231 cell line^1,22^, providing a plausible candidate for the driving force of the attraction via computer model we will discuss next.

## Cellular Potts Model

**Figure 5:**
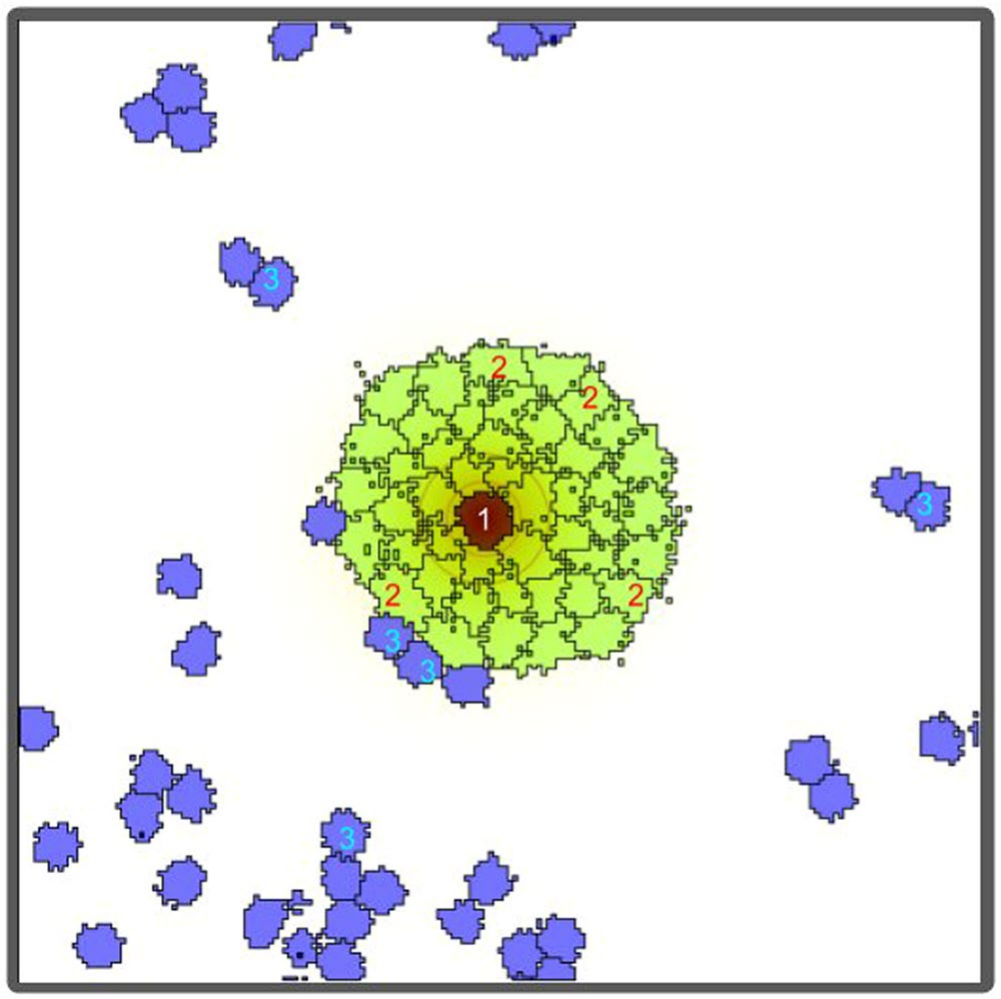
Schematic illustration of different domains introduced in our Potts Model. All together there are four different 3D domains: one core region of a senescent cell (indexed by *σ* = 1, target volume = 1800 *μ*m^3^, target surface asperity = 0.8); a tightly connected aggregate of 48 small domains (*σ* = 2, target volumes = 600 *μ*m^3^, target surface asperity = 0.8) maintaining a very thin, yet, dynamic cytoskeletal structure around the core; 20 small domains (*σ* = 3, target volume = 400 *μ*m^3^, target surface asperity = 1.4) representing non-senescent tumor cells; and, one simply-connected domain (*σ* = 0) representing the culture medium the cell domains are immersed in. The shown 2D image is a 2D slice (n = 2) of 3D z-stack images. Only a few domains are labeled for clarity.

The observed cluster formation could be recapitulated by a modified CPM. The original CPM was developed by Chiang *et al*.^23^ to elucidate the phenomenon of cell sorting. CPM treats cells as simply-connected domains in 2D (volumes in 3D) in a grid lattice whose evolution is determined by Monte Carlo simulation and Metropolis algorithm. The Hamiltonian embedded in our model takes into account three major factors; the conservation of a cell’s surface area and volume, varying surface adhesion between different types of cells, and chemotactic motion. From the perspective of our CPM, there are co-evolving 4 different types of 3D domains (Fig. 5): 1 core region of a senescent cell (indexed by *σ* = 1); a tightly connected aggregate of 48 small domains (*σ* = 2) maintaining a very thin, yet, dynamic cytoskeletal structure (body) around the core; (initially) 20 small domains (*σ* = 3) representing non-senescent tumor cells which replicate with a period *τ*_*r*_ = 5000 ± 1000 s (mean ± sd); and, one simply connected domain (*σ* = 0) representing the culture medium into which the cell domains are immersed for the 2D graphical illustration of these domains).

One key rule that we adopted to capture our observed phenomenon is the dynamic surface adhesiveness accompanied by mitotic cell-rounding, during which the cell’s adhesion to the environment significantly weakens upon division (a more detailed implementation of the adhesion loss given in Methods). This idea is reminiscent of the calcium-dependent modulation of cell adhesion in the CPM built by Czirók *et al*.^24^. Another important property is the field, *ω*, acting as a chemo-attractant (or one of SASPs) released by the senescent cell’s core. *ω* diffuses to its vicinity (to the body itself, and the empty space), and degrades in time: It obeys $\frac{\partial \omega }{\partial t}=p-d\omega +D{\nabla }^{2}\omega$, where *p*, *d* and *D* represent the rate of *ω* production, decay rate and diffusion coefficient, respectively. Finely tuning a cell’s sensitivity to the chemo-attractant allowed only the newly divided cells to climb on senescent cell’s body, while non-dividing cells are obstructed by the tightly joined body domain (bright green region in Fig. 5). A detailed description on the parameters used in the model is given in Methods.The simulation was performed in the CPM simulation platform, Morpheus^25^.

The cellular Potts model constantly changes configuration of the system based on the thermodynamic principle which is summarized to the fact that a system prefers lower-energy condition. Every lattice site is assigned to a cell type (4 in our system; senescence core, senescence body, normal cell, and the medium). The probability of a target site changes its type to that of one of its immediate neighbors is given by Boltzmann probability,

$${p}_{copy}=(\begin{array}{cc}\mathrm{1,} & {\rm{if}}\,{\rm{\Delta }}{H} < {\rm{0}}\\ {e}^{-{\rm{\Delta }}H/T}, & {\rm{otherwise}}\mathrm{.}\end{array}$$

where *p*_*copy*_ means the probability that the target site copies the chosen cell type. The change of free energy, Hamiltonian, is calculated between two systems with the current cell type and the copied cell type at the target site. *T* represents the ‘Temperature’ which determines the level of fuzziness of a cell’s boundary. The Hamiltonian in our simulation is comprised of three essential parts which drive the whole process.

$$\begin{array}{ccc}{H}_{constraints} & = & \sum _{\sigma }[{\lambda }_{V}{({v}_{\sigma }-{V}_{t})}^{2}+{\lambda }_{P}{({P}_{\sigma }-{P}_{t})}^{2}]\\ {H}_{interaction} & = & \sum _{{\rm{interface}}(i,j)}J({\tau }_{{\sigma }_{i}},{\tau }_{{\sigma }_{j}})(1-{\delta }_{{\sigma }_{i}{\sigma }_{j}})\\ {\rm{\Delta }}{H}_{chemotaxis} & = & \sum _{\sigma =3}\mu (\omega (x^{\prime} )-\omega (x))\end{array}$$

where the sum over the index, *σ*, indicates summing over all the lattice sites of the designated cell type. *H*_*constraints*_ places constraints on a cell’s volume and surface area (*V*_*t*_, *P*_*t*_ are target volume and surface area, or perimeter in 2D, respectively). *H*_*interaction*_ applies cell-cell interaction energy, and is added along the entire intersection between differing cell types. Lastly, Δ*H*_*chemotaxis*_ puts chemotactic influence to the normal cells (*σ* = 3). It depends upon the difference in the intensity of the field at the target site, *ω*(*x*), and at the neighboring site, *ω*(*x*′). ${\lambda }_{V},{\lambda }_{P},J({\tau }_{{\sigma }_{i}},{\tau }_{{\sigma }_{j}})$, and *μ* respectively represent volume, and perimeter elasticity, surface energy, and the level of sensitivity to the chemo-attractant.

**Figure 6:**
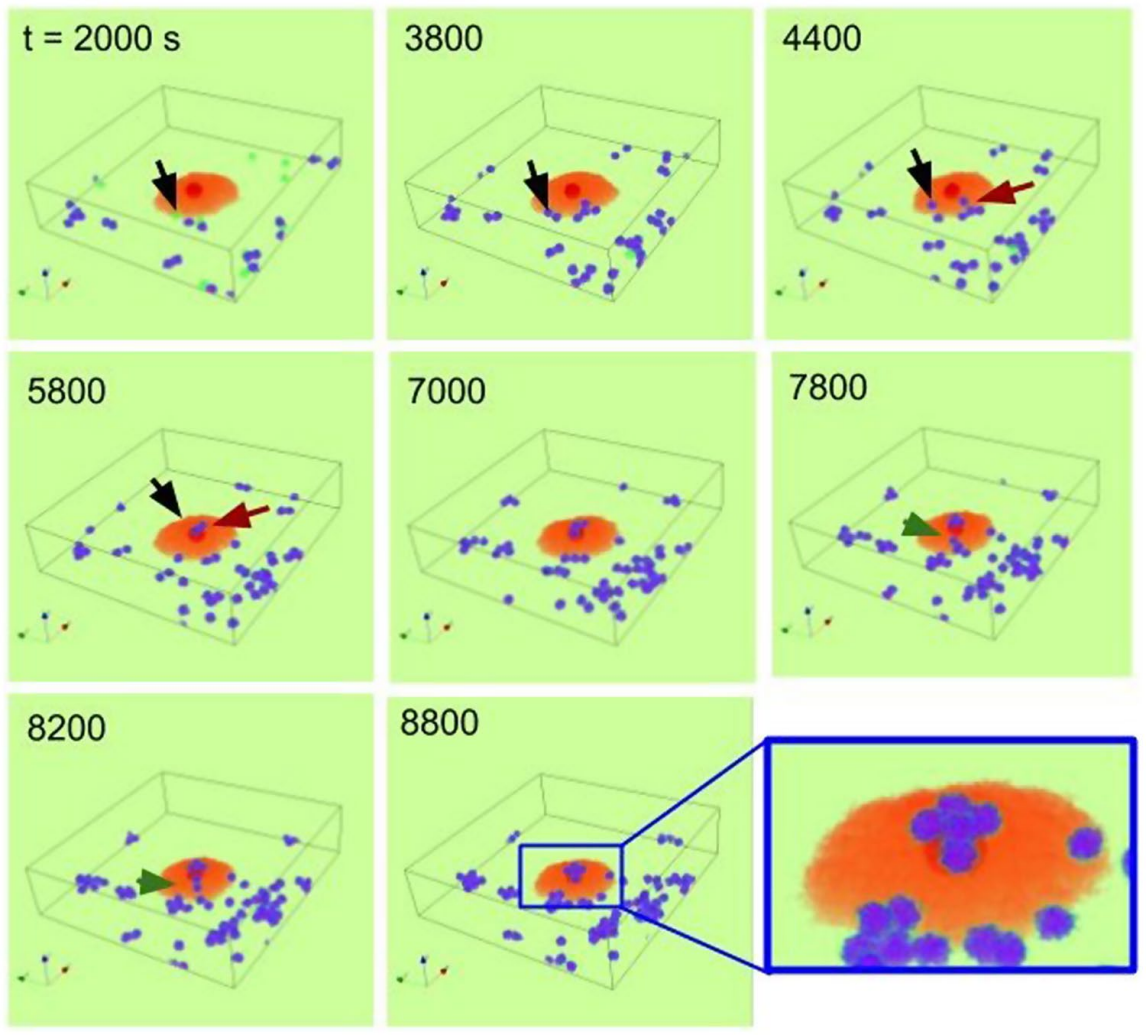
3D simulation of a cell cluster formation at the core of a senescent cell. 1*s* is equivalent as one Monte Carlo step. The black and brown arrows mark the positions of two single (separated) daughter cells from *t* = 2000 s up to *t* = 5800 s. Similarly, the green arrow-heads mark the position of one ‘dumbbell’ (a pair of daughter cells) from *t* = 7800 s up to *t* = 8200 s. The marked daughter cells migrate towards the core to form a 3D cluster (see Supplementary Video S3). The grid size is 200 × 200 × 50 *μ*m^2^, and periodic boundary conditions are applied at all boundaries except for the top and the bottom. Non-senescent cells replicate with a period *τ*_*r*_ = 5000 ± 1000 s (mean ± sd). The following color code scheme is used: senescent cell (core: dark red, body: light red); first generation tumor cells (green), replicated tumor cells (blue). *p* = 2 *μm*^−3^*s*^−1^, *d* = 0.02 *s*^−1^ and *D* = 2 *μ*m^2^*s*^−1^. The rest of parameter values are given in Methods.

Figure 6 illustrates a time-sequence of snapshot images of the simulation. We defined ‘1*s*’ as one Monte Carlo step during which the entire lattice site is updated. The ‘fried-egg’ shape is well reflected into the model whose structure consists of a bulged core (dark red) and a flat body (light red). Tumor cells that were divided at least once are colored blue, while cells that have never gone through a replication are colored green. For example, the green cell, which is marked by a black arrow in the frame of *t* = 2000 s, is doubled to form a dumbbell-like structure at *t* = 3800 s. Then, one of the two daughter cells slowly migrates toward the core region. Another daughter cell originating from a different position, marked by a brown arrow at *t* = 3800 s, also migrates towards the core region to join the previous one forming a pair of tumor cells at the core (see *t* = 5800 s). The cells aggregated at the core can also replicate (see *t* = 7000 s). Moreover, just like in the experiment, the daughter cells often migrate in a form of dumbbell as well illustrated in the sequence of snapshot images from *t* = 7800 s to *t* = 8800 s (highlighted by green arrow heads). As a consequence, a small 3D cell cluster is formed just above the core region of the senescent cell (see the inset of *t* = 8800 s and Supplementary Video S3).

## Discussion

Growing population of cells cultured on a low-attachment plate often renders 3D clusters of cell as in the preparations of tumor spheroids^26^ or cardiac spheroids^27^. The processes involve cellular differentiations and tissue structural changes that are subject to cell motility and sorting, all of which are driven by a complex set of biochemical and biophysical events. Likewise, the underlying mechanism for the 3D cell cluster formation of MDA-MB-231 cells that we report here may require the same level of complexity for a full understanding. Nonetheless, the observed phenomenon could be successfully recapitulated by a simple CPM that is solely based on a Hamiltonian, of which the kinetic energy (associated with chemotactic force) competes with interface energies (associated with interface adhesions). Similar to any other CPM approaches^23,24^, the novelty of our CPM is its simplicity including only the key biophysical processes for the given phenomenology, namely, chemotactic attraction, time-varying interface adhesion associated with mitotic cell rounding, and relative strengths of adhesion [as in the case of Steinberg’s well-known explanation of cell-sorting]^28^. In order to reproduce the experimental observation, a set of parameters is chosen such that the chemotactic force is strong enough only for the newly divided cells adjacent to a senescent cell to have directed migration. In fact, we can tune various parameters of the model system to study many related issues like the role of “neighborhood crowding,” just for an example. Besides, the current CPM can be extended to a fully 3D tissue for studying 3D structural deformation associated with dynamic evolution of senescent cells in conjunction with proliferating non-senescent cells around them.

The most unusual finding is that 3D cell cluster formations are initiated and organized actively by senescent cells. Similar to the developmental process of starving Dd amoeba on a culture plate?, the focal points of each aggregation site are nothing but some randomly chosen cells, which have gone through cellular senescence, among the same cell population. For the case of Dd amoebae, the focal points are often called as a pacemaker as it is releasing chemo-attractants cAMP in a periodic fashion. Subsequently, Dd amoebae chemotaxis towards their nearest pacemaker in a pulsatile fashion while relaying the cAMP signal actively to neighboring cells by excitable waves^29^. Unlike the case of chemotactic Dd amoeba, the MDA-MB-231 cells migrating towards the core of senescent cell neither show any pulsatile movements nor seem to relay the chemical signal for chemotaxis: This suggests that chemo-attractants are released in a continuous fashion from the core of senescent cell and they only diffuse out, and subsequently a diffusion-mediated short-range chemotaxis is incorporated into our CPM.

At this point, we should point out that non-trivial 3D structures (eg. cell clusters, large-scale collective waves or swirls, instead of uniform 3D layer) can form in a proliferating cell population even in the absence of senescent cells with different mechanisms^19,24^. We have not investigated that possibility carefully with our MDA-MB-231 cultures. As aforementioned, what we have observed is that senescent MDA-MB-231 cells in the population serve as a source of premature multi-layer transition and importantly population confluency is not a requirement for the formation of 3D cell clusters.

## Conclusion

In summary, we have discussed an unusual observation as to the senescent cancer cell’s role as a source of colonial transition to a multi-layered cell colony. We view that this is a robust *in vitro* demonstration that cellular senescence can play a crucial role in tissue restructuring. As for the underlying mechanism of the directed cell movements towards the senescent cell’s nucleus, we have proposed chemotaxis-driven cell motility via computer simulation. However, more investigation is needed to identify the exact kind and nature of the responsible chemo-attractant. Finally, cellular senescence, in general, is a phenomenon found in numerous cell types and tissue environments, although its abundance of occurrence must depend on the micro-environment. Accordingly, we speculate that the observed senescence-mediated cell aggregation and the consequent tissue rearrangement can occur in a wide spectrum of biological contexts including conditions with higher topological complexity. The consequence could be as detrimental as the promotion of tumor spread, or provide a vital contribution in recovering from it^30^.

## Methods

### Cell culture and sample preparation

Confluent colony of MDA-MB-231 breast cancer cells on cover glass were fixed in 4% paraformaldehyde and 4% sucrose at room temperature for 20 min. It was washed three times with 1X Tris-Buffered Saline (TBS) on the 3D Rocker for 10 min each. Blocking was done with blocking solution (3% BSA, 0.2% Triton X-100 in TBS) on the 3D Rocker for 30 min. Primary antibodies (E-cadherin - 1:500, phalloidin 1:50 in the blocking solution) was applied at 4 °C overnight. Then, it was washed three times with TBS-T solution (0.05% Tx-100 in 1X TBS) for 10 min each. E-cadherin antibody, as a secondary antibody, was diluted 1:500 in blocking solution and stored with the cell for 30 min (for a plate, shaking on the 3D Rocker). First wash was done with TBS-T for 10 min and second wash was done with TBS-T solution containing Hoechst (diluted 1:2000 in TBS-T) on the 3D Rocker for 10 min. Final wash was done again with TBS-T for 10 min. Aqueous mounting medium was applied to solidify the sample. The antibodies used target f-actin (488 phalloidin, green), E-cadherin (Cy3, or red) and nuclei (Hoechst, blue). A confocal microscopeimaging-device was used to capture the sample image (TCS-SP8, Leica).

### Immunocytochemistry

Confluent colony of MDA-MB-231 breast cancer cells on cover glass were fixed in 4% paraformaldehyde and 4% sucrose at room temperature for 20 min. It was washed three times with 1X Tris-Buffered Saline (TBS) on the 3D Rocker for 10 min each. Blocking was done with Blocking solution (3% BSA, 0.2% Triton X-100 in TBS) on the 3D Rocker for 30 min. Primary antibodies (E-cadherin 1:500, phalloidin 1:50 in the blocking solution) was applied at 4 C overnight. Then, it was washed three times with TBS-T solution (0.05% Tx-100 in 1x TBS) for 10 min each. E-cadherin antibody, as a secondary antibody, was diluted 1:500 in blocking solution and stored with the cell for 30 min (for a plate, shaking on the 3D Rocker). First wash was done with TBS-T for 10 min and second wash was with Hoechst (diluted 1:2000 in TBS-T) on the 3D Rocker for 10 min. Final wash was done again with TBS-T for 10 min. Aqueous mounting medium was applied to solidify the sample. The antibodies target f-actin (488 phalloidin, green), E-cadherin (Cy3, or red) and nuclei (Hoechst, blue). A confocal microscope/imaging-device was used to capture the sample image (TCS-SP8, Leica).

### Time-lapse imaging and analysis

A small thin cylindrical incubator was designed and lab-built for long-term live-cell imaging. An insulated heating pad heated the distilled water inside the incubator, and the temperature inside was monitored by a thermometer (PT100, Sanup electronics, Korea) and actively regulated at 37.5 ± 0.1 °C by a temperature controller (SDM9000, Sanup, Korea). Two ITO-coated optical windows of the incubator along the imaging axis were also electrically heated. Gas mixture (5% CO_2_, 95% air) was continuously bubbled in through a water tank.

A sample petri-dish containing a defined MDA-MB-231 cell layer was loaded into the incubator, which was then placed on the stage of an inverted microscope (IX71, Olympus) with 4 × (NA 0.13) and 10 × (NA 0.30) objective lenses. Time-lapse phase-contrast images were acquired with time intervals between 15 s or 30 s, typically for 1~4 days using ProgRes MFcool CCD Camera (Jenoptik, Germany). The camera has a sensor resolution of up to 1360 × 1020 pixels with each pixel size of 6.45 × 6.45 *μ*m^2^.

### Parameters used in the CPM simulation

The following set of interaction energies (adhesiveness) are used: ${J}_{\mathrm{core},\mathrm{body}}=-\,170$; ${J}_{\mathrm{core},\mathrm{tumor}}=-\,10$; ${J}_{\mathrm{core},\mathrm{medium}}=10$; ${J}_{\mathrm{core},\mathrm{matrix}}=-\,70$; ${J}_{\mathrm{body},\mathrm{medium}}=4$; ${J}_{\mathrm{body},\mathrm{body}}=-\,120$; ${J}_{\mathrm{body},\mathrm{tumor}}=-2$; ${J}_{\text{tumor},\text{tumor}}=-\,6$; ${J}_{\mathrm{tumor},\mathrm{medium}}=-\,4$; ${J}_{\text{tumor},\text{matrix}}=-\,20$; and ${J}_{\mathrm{medium},\mathrm{matrix}}=10$, where the subscripts, *core*, *body*, *tumor*, *matrix* and *medium* represent the core portion of senescent cell, the compartmentalized domains representing the thin body of senescent cell, the non-senescent tumor cells, the substrate and culture medium, respectively. In order to simulate the weaker tumor cell to tumor cell adherence during the mitotic cell rounding, we let the interaction energy of a tumor cell with its neighboring tumor cells switch from ${J}_{\text{tumor},\text{tumor}}=-\,6$ to $-\,6-\,110{e}^{-(t-{t}_{0}\mathrm{)/100}}$ at the onset *t* = *t*_0_ of each cell replication. At the same time, we also change the interaction energy of the replicating tumor cell with the substrate from ${J}_{\text{tumor},\text{matrix}}=-\,20$ to $-\,20-\,110{e}^{-(t-{t}_{0}\mathrm{)/500}}$. Both interaction energies gradually recover to the resting value of −6 and −20 with a time constant of 100 and 500, respectively.

Metropolis kinetics with temperature *t* = 100.0 and interaction neighborhood order of 2 are used for the simulation. One second is equivalent to one Monte Carlo time step that represents an update for all grid values.

## Electronic supplementary material

supplementary information

Video S1

Video S2

Video S3
