# Supplementary material for: Senescent tumor cells building three-dimensional tumor clusters
Source: Sci Rep. 2018 Jul 12;8:10503. doi: 10.1038/s41598-018-28963-0 (PMC6043561; doi:10.1038/s41598-018-28963-0)
Supplement: Supplementary file 1 — supplementary information [file 41598_2018_28963_MOESM1_ESM.pdf]

Senescent tumor cells building three-dimensional tumor clusters: supplementary material

Hyun-Gyu Lee[1], June Hoan Kim[2], Woong Sun[2], Sung-Gil Chi[3], Wonshik Choi[1,4], and Kyoung J. Lee[1*]

[1]Department of Physics, Korea University, Seoul 02841, Korea
[2]Department of Anatomy, Korea University College of Medicine, Seoul 02841, Korea
[3]Department of Life Sciences, Korea University, Seoul 02841, Korea
[4]Center for Molecular Spectroscopy and Dynamics, Institute for Basic Science, Seoul 02841, Korea
[*]kyoung@korea.ac.kr

1 Supplementary figures

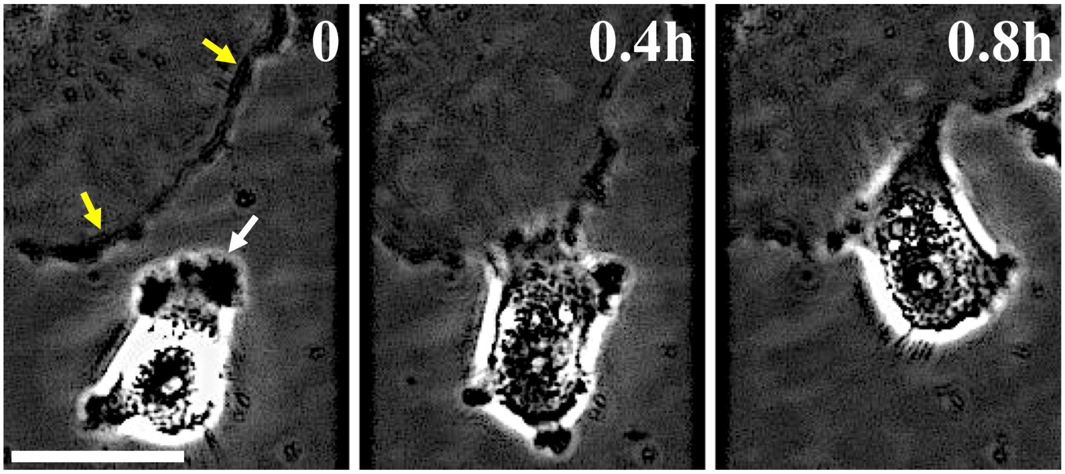

Figure S1 Contact inhibition of movement of non-senescent MDA-MB-231 cell facing the cell boundary of a senescent MDA-MB-231 cell. The active ruffle dynamics of both cell before the contact (see the frame at time 0 h, white arrow: leading lamella of non-senescent cell; yellow arrows: ruffles at the boundary of a senescent cell) cease where the contact sets in (see frames at 0.4 and 0.8 h). Normally, the non-senescent cell does not crawl over the dorsal surface of the senescent cell body. [scale bar: 25 μm]

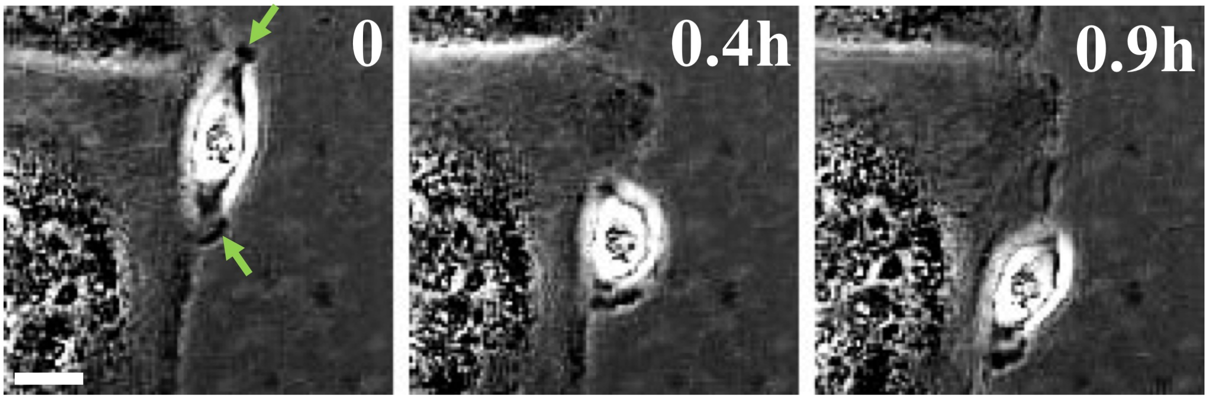

Figure S2 Tangential moment of a non-senescent MDA-MB-231 cell along the boundary of a senescent MDA-MB-231 cell. Membrane protrusions and ruffles (marked by green arrows) exist mostly in the direction of movement. As in a typical contact inhibition, ruffles are not supported at the moving contact line. [scale bar: 25 μm]

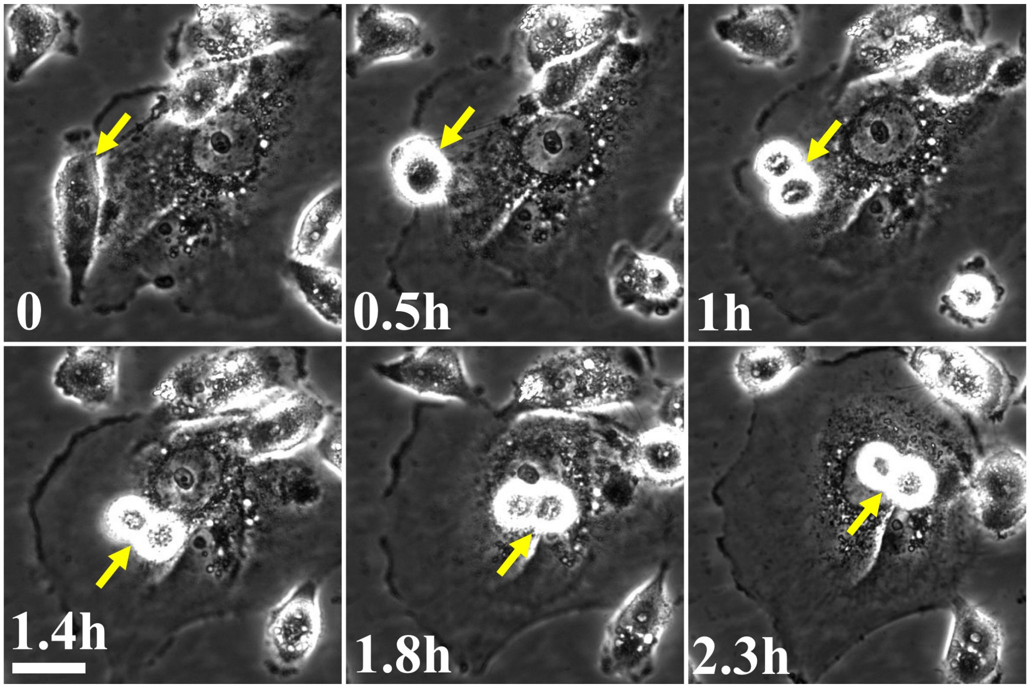

Figure S3 A sequence of snapshot images showing a divided MDA-MB-231 daughter cell (marked by a yellow arrow), in a non-crowded environment, migrating towards the core of a neighboring senescent cell. This sequence of images illustrates the point that population crowding is not an essential factor for the 3D cell cluster formation at the core of senescent cell body. [scale bar: 25 μm]

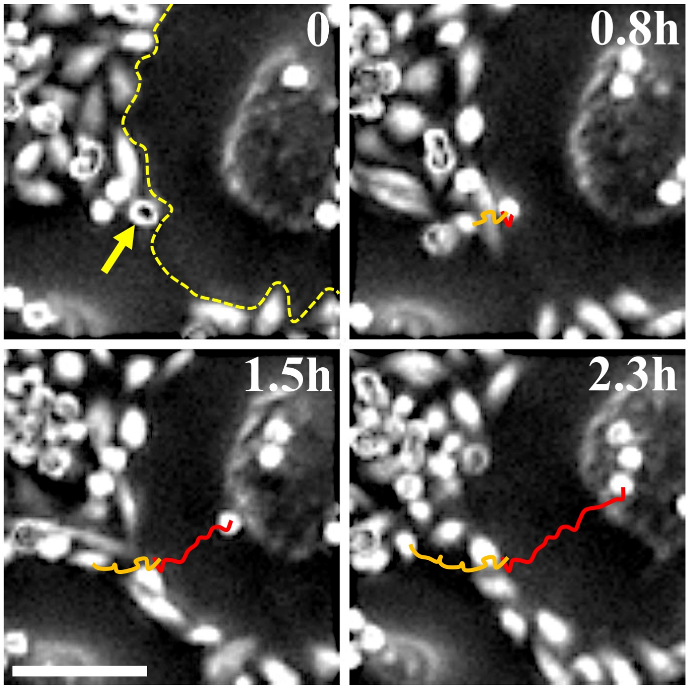

Figure S4 A sequence of snapshot images illustrating a case in which only one (marked in red) of divided pair of MDA-MB-231 cell migrates towards the senescent cell core while the other (marked in yellow) is reuptaken into the substrate. [scale bar: 100 μm]

2 Legends for Supporting Videos

Video S1 Movie showing non-senescent MDA-MB-231 tumor cells moving along the boundary of an adjacent senescent MDA-MB-231 cell (see Fig. 2d).

Video S2 Movie showing newly replicated MDA-MB-231 daughter cell pairs detach themselves from the surroundings and migrate towards the core of adjacent senescent cell (see Fig. 4)

Video S3 Numerical simulation showing newly replicated CPM cells detach themselves from the surroundings and migrate towards the core of adjacent senescent cell (see Fig. 6)
